# Effects of dietary supplementation with a combination of plant oils on performance, meat quality and fatty acid deposition of broilers

**DOI:** 10.5713/ajas.18.0056

**Published:** 2018-04-12

**Authors:** Shenfei Long, Yetong Xu, Chunlin Wang, Changlian Li, Dewen Liu, Xiangshu Piao

**Affiliations:** 1 State Key Laboratory of Animal Nutrition, China Agricultural University, Beijing 100193, China; 2 Shandong Zhongda Agriculture Science and Technology Co. Ltd., Binzhou 256600, China

**Keywords:** Broiler, Fatty Acids, Meat Quality, Mixed Oils, Performance

## Abstract

**Objective:**

This study was to evaluate effects of mixed plant oils (identified as mixed oil 1 [MO1] and mixed oil 2 [MO2]) on performance, serum composition, viscera percentages, meat quality, and fatty acid deposition of broilers.

**Methods:**

A total of 126 one-day-old Arbor Acres male broiler chicks (weighing 44.91± 0.92 g) were randomly allocated to 1 of 3 treatments with 7 replicate pens per treatment (6 broilers per pen). Dietary treatments included a corn-soybean basal diet supplemented with 3% soybean oil (CTR), basal diet with 3% MO1 (a mixture of 15% corn oil, 10% coconut oil, 15% linseed oil, 20% palm oil, 15% peanut oil and 25% soybean oil; MO1), or basal diet with 3% MO2 (a combination of 50% MO1 and 50% extruded corn; MO2). The trial consisted of phase 1 (d 1 to 21) and phase 2 (d 22 to 42).

**Results:**

Compared to CTR, broilers fed MO (MO1 or MO2) had greater (p<0.05) average daily gain in phase 1, 2, and overall (d 1 to 42), redness in thigh muscle, concentrations of serum glucose, serum albumin, saturated fatty acids (SFA) and n-6/n-3 polyunsaturated fatty acids (PUFA) ratio in breast muscle, while these broilers also showed lower (p≤0.05) drip loss and concentrations of C18:3n-3 and PUFA/SFA ratio in breast muscle. Broilers fed MO2 had higher (p<0.05) liver percentage, while broilers fed MO1 had lower (p≤0.05) feed conversion ratio in phase 1 and increased (p<0.05) contents of C18:2n-6, C20:5n-3, C22:6n-3, and n-3 PUFA in breast muscle compared to CTR.

**Conclusion:**

Mixed plant oils had positive effects on performance, serum parameters, meat quality, liver percentage and fatty acid deposition in broilers, which indicates they can be used as better dietary energy feedstocks than soybean oil alone.

## INTRODUCTION

Oil is often used as a high energy feedstock in animal diets. Broilers fed rations containing oil show better performance and higher digestibility of fat than those fed diets without oil inclusion although the nutritive value of the diets is similar [1]. The fatty acid profile and carcass quality of poultry meat is influenced by the fatty acid profile of the diet consumed by the broilers during rearing [2]. Among all the oil feedstocks, corn oil, coconut oil, linseed oil, palm oil, peanut oil and soybean oil can be widely used in broilers’ diet. Corn oil is rich in oleic acid and linoleic acids, while 4% corn oil can improve performance and meat quality of broilers [3]. Coconut oil can reduce fat deposition and favorably affect lipid profiles without impairing performance in broilers [4]. Linoleic and linolenic fatty acids derived from linseed oil are recognized as metabolically essential for broilers because broilers are not able to synthesize these fatty acids, and linoleic acid is the only essential fatty acid whose diet requirement has been demonstrated [5,6]. Broilers fed a diet containing 2% linseed oil showed increased total polyunsaturated fatty acids (PUFA), decreased total monounsaturated fatty acids proportions and improved n-6/n-3 PUFA ratio in breast muscle of broilers [7]. Soybean oil, the most common oil feedstocks utilized in broilers diets, can improve performance and the local immune system in the small intestine of broilers [8,9]. Although these plant oil feedstocks have beneficial effects on broilers, the mixed effect of them remains to be investigated.

Many previous studies have showed that a combination of oil feedstocks may be more beneficial for performance of broilers than single oil feedstock [10]. Broilers fed a diet supplemented with a combination of tallow, sunflower oil, and linseed oil showed improved performance and fatty acid profile in the breast and thigh meat [11]. Besides, a combination of beef tallow, olive oil, sunflower oil, and linseed oil in broiler diets could produce less fat deposits than the ones rich in saturated or monounsaturated fatty acids [12]. The differences in dietary fatty acid absorption may be due to long-chain unsaturated fatty acids having greater ability to form micelles and can act synergistically in the absorption of saturated fatty acids (SFA) when mixed [13]. Therefore, a study of the effects of a combination of different kinds of vegetable oils containing both unsaturated fatty acids and SFA would be valuable.

Therefore, the objective of this study was to investigate the effects of two novel mixed plant oils containing a combination of corn oil, coconut oil, linseed oil, palm oil, peanut oil, and soybean oil, in comparison to soybean oil alone, on performance, serum parameter, meat quality, viscera percentages and fatty acid deposition in broilers.

## MATERIALS AND METHODS

All the procedures used in the animal experiment were approved by the Institutional Animal Care and Use Committee of China Agricultural University (Beijing, China).

### Experimental products

The source of dietary mixed oils used in this study were prepared and supplied in Shandong Zhongda Agriculture Science and Technology Co. Ltd. (Binzhou, China). Chemical composition of mixed oil 1 (MO1) and mixed oil 2 (MO2) is summarized in Table 1. The MO1 is a combination of 15% corn oil, 10% coconut oil, 15% linseed oil, 20% palm oil, 15% peanut oil, and 25% soybean oil, while MO2 is a mixture of 50% MO1 and 50% extruded corn. Concentrations of palmitic acid (C16:0), oleic acid (C18:1n-9), linoleic acid (C18:2n-6), and α-linolenic acid (C18:3n-3) in MO1 are 17.4%, 17.5%, 36.3%, and 2.50%, while concentrations of palmitic acid, oleic acid, linoleic acid, and α-linolenic acid (C18:3n-3) in MO2 are 9.79%, 10.2% 21.7%, and 1.49%, respectively. The formulations of experimental diets used in this study are summarized in Table 2.

### Experimental animals and management

A total of 126 one-day-old as-hatched Arbor Acres chicks (weighing 44.91±0.92 g) were purchased from Arbor Acres Poultry Breeding Company (Beijing, China). All broilers were raised in wire-floor cages in an environmentally controlled room with continuous light (10 to 20 lux) and had *ad libitum* access to feed and water. The room temperature was maintained at 33°C for the first 3 d, then the temperature was reduced by 3°C a week until reaching 24°C and this temperature was maintained until the end of the 42-d experiment. The lighting regimen and ventilation were monitored continuously from d 1 to d 42. All broilers were inoculated with Newcastle disease vaccine on d 7 and d 28 and with inactivated infectious bursa disease vaccine on d 14 and d 21.

### Experimental design and diets

The broilers were randomly allocated to 1 of 3 treatments. Dietary treatments included a corn-soybean basal diet supplemented with 3% soybean oil (CTR), basal diet supplemented with 3% MO1 (MO1), and basal diet supplemented with 3% MO2 (MO2). There were 7 replicate pens per treatment, with 6 broilers per pen. The study was conducted over phase 1 (d 1 to d 21) and phase 2 (d 22 to d 42). All essential nutrients in the basal diet met the requirements suggested by NRC [5]. All diets were fed as mash form.

### Processing procedure

On d 21 and 42, the broilers were fasted for 12 h and the broilers and feeds were then weighed to determine average daily gain (ADG), average daily feed intake, and feed conversion ratio (FCR). The diets were ground to pass through a 1-mm sieve before analysis, and were analyzed according to the procedures of the Association of Official Analytical Chemists [14]. The diets were tested for crude protein (CP, method 990.03; AOAC [14]). The mixed oils were analyzed for CP, dry matter (method 934.01; AOAC [14]), ash (method 942.05; AOAC [14]), extract ether (method 920.39; AOAC [14]), while the gross energy was determined by an automatic isoperibolic oxygen bomb calorimeter (Parr 1281, Automatic Energy Analyzer; Moline, IL, USA).

On d 21 and d 42, one broiler per pen (weighing closest to the average body weight in each pen, n = 7) was selected and euthanized for blood samples. Blood was collected (5 mL) by cardiac puncture into a 10-mL anticoagulant-free Vacutainer tube (Greiner Bio-One GmbH, Kremsmunster, Austria) and centrifuged at 3,000 g for 10 min to obtain serum. Serum samples were stored at −80°C until needed for analysis.

### Determination of meat quality and viscera percentages

The broilers weighting close to average body weight from each pen (n = 7) on d 42 were also used to determine meat quality. The left breast and thigh muscle were removed and weighed. Meat color, including lightness (L^*^), redness (a^*^), and yellowness (b^*^) values, was measured from 3 orientations (middle, medial, and lateral) using a Chromameter (CR-410, Konica Minota, Tokyo, Japan). Postmortem drip loss at 24 h was measured using the plastic bag method as described previously [15]. On d 42, the visceras of broilers, including heart, liver, spleen, pancreas and abdominal fat, were collected and weighed to determine the viscera percentages (viscera percentages = viscera weight/final body weight×100%).

### Determination of serum indices

The concentrations of glucose, globulin, albumin, albumin/globulin ratio, total protein, total cholesterol (TC) and high-density lipoprotein cholesterol in serum samples were analyzed by an automatic biochemical analyzer (RA-1000, Bayer Corp., Tarrytown, NY, USA) using colorimetric methods, following the instructions of the manufacturer of the corresponding reagent kit (Zhongsheng Biochemical Co., Ltd., Beijing, China).

### Determination of fatty acid profile

Homogenized skinless thigh meat tissue of broilers (weighting close to average body weight from each pen, n = 7) were defrosted and samples of breast meat (ca. 20 g) lyophilized for 60 h using a freeze dryer. Fatty acid concentrations of lyophilized d 42 breast meat and milled feed was determined. The fatty acid profiles of the lipid sources were determined by gas chromatography (6890 series, Agilent Technologies, Wilmington, DE, USA) according to the procedures of Sukhija and Palmquist [16] with slight modifications. Lipid samples were converted to fatty acid methyl esters using methanolic HCl. Undecanoic acid (C11:0) was used as the internal standard. Aliquots of 1 mL were injected into a capillary column (60 m×250 m×250 nm, DB-23, Agilent, USA) with cyanopropyl methyl silicone as the stationary phase. Column oven temperature was programmed with a 1:20 split. Injector and detector temperatures were maintained at 260°C and 270°C, respectively. Nitrogen was the carrier gas at a flow rate of 2 mL/min. Fatty acid composition were then calculated in terms of milligrams of fatty acid per 100 g of tissue (fresh weight).

The data of SFA, PUFA, n-6 PUFA, n-3 PUFA, and PUFA/SFA ratio (P/S) were calculated values, the statistical formula is as follows:

$$\begin{array}{l} \text{N-}6\;\text{PUFA:}\;\text{C}18:2\text{n-}6+\text{C}18:3\text{n-}6+\text{C}20:4\text{n-}6\\ \text{N-}3\;\text{PUFA:}\;\text{C}18:3\text{n-}3+\text{C}20:5\text{n-}3+\text{C}22:6\text{n-}3\\ \text{SFA:}\;\text{C}14:0+\text{C}16:0+\text{C}17:0+\text{C}18:0+\text{C}20:0\\ \text{PUFA:}\;\text{C}18:2\text{n-}6+\text{C}18:3\text{n-}6+\text{C}20:4\text{n-}6+\text{C}18:3\text{n-}3+\text{C}20:5\text{n-}3+\text{C}22:6\text{n-}3 \end{array}$$

### Statistical analyses

Data were subjected to analysis of variance using the general linear model procedure of SAS (SAS Institute, Raleigh, NC, USA). The pen was the experimental unit. Differences among treatments were separated by Duncan’s multiple range test. Results were expressed as least squares means and standard error of the mean. Significance was designated at p≤0.05, while a tendency for significance was designated at 0.05<p≤0.10.

## RESULTS

### Broiler performance

The effects of MO on performance of broilers are summarized in Table 3. Compared to CTR, broilers fed MO (MO1 or MO2) had greater (p<0.05) body weight on d 21 and d 42, as well as increased (p<0.05) ADG during phase 1, 2 and overall (d 1 to d 42), while broilers fed MO1 showed lower (p≤0.05) FCR in phase 1.

### Meat quality and viscera percentages

The effects of MO on meat quality and viscera percentages of broilers are shown in Table 4. Broilers fed MO2 had higher (p<0.01) liver percentage, while broilers fed MO showed greater (p<0.01) redness in thigh muscle compared to CTR. Broilers fed MO1 had a tendency for a decrease of yellowness in breast and thigh muscle, while drip loss in breast and thigh muscle was decreased (p≤0.05) in broilers fed MO compared to CTR.

### Serum parameters

The effects of MO on serum parameters of broilers are presented in Table 5. During phase 1, broilers fed MO1 showed a tendency for an increased (p = 0.08) concentration of glucose in serum compared to CTR. In phase 2, broilers fed MO showed higher (p≤0.05) concentration of glucose and albumin, as well as a tendency for increased contents of globulin and total protein in serum compared to CTR; whereas broilers fed MO1 showed a tendency (p = 0.10) for a decreased concentration of TC in serum compared to CTR.

### Fatty acids in breast muscle

The effects of MO on fatty acids deposition in breast muscle of broilers are shown in Table 6. Broilers fed MO had increased (p<0.05) concentrations of C14:0, C16:0, C17:0, C18:0, C20:0, C20:4n-6, SFA, and n-6/n-3 ratio; whereas these broilers also showed decreased (p<0.05) concentrations of C18:3n-3 and P/S ratio in breast muscle compared to CTR. Broilers fed MO1 had increased (p<0.05) contents of C18:2n-6, C20:5n-3, C22:6n-3, n-6 PUFA, n-3 PUFA, and total PUFA, as well as a tendency for a lower concentration (p = 0.06) of C16:1n-7 in breast muscle compared to CTR.

## DISCUSSION

In this research, broilers fed MO showed greater ADG and decreased FCR than those fed soybean oil alone, which is somewhat consistent with Crespo and Esteve-Garcia [2], who reported that broilers fed a diet containing a combination of sunflower and linseed oil showed lower FCR. This result might be due to the beneficial effects of an appropriate balance between SFAs (e.g. 20% palm oils) and PUFAs (e.g. 15% linseed oil) [10]. Moreover, since MO is rich in palmitic acid, oleic acid, linoleic acid and α-linolenic acid, the reason for these improvements of performance can also be the high concentrations of oleic acid and linoleic acid in MO that helped to improve oil utilization and eventually increase ADG and decrease FCR in broilers [17].

Along with the improved performance, broilers fed MO also showed improved viscera percentages and meat quality. The normal development of animal viscera is the basis of the physical function of animal body, which indicates greater viscera percentages may reflect better viscera metabolism and function. In broilers, liver is the most important fat metabolism organ because more than 95% of fat synthesis occurs in the liver [18]. Therefore, the higher liver percentage in broilers fed MO2 in the present study reveals greater fat synthesis in these broiler. Color is used as an indicator of freshness, quality and economic value of broiler meat. The unattractive brown color (yellowness) and decreased redness value in meat may be caused by oxidation of red oxymyoglobin to metmyoglobin [19]. In the present study, broilers fed MO showed increased redness value and a tendency for a reduced yellowness in meat, which indicates MO can help to minimize the oxidation of myoglobin, maintain meat color of broilers, and minimize the occurrence of pale, soft, and exudative meat in broilers [20]. The increased redness in thigh muscle also reflects the increased growth of muscle fibers, which can help to improve meat quality in broilers [21]. There is a strong negative correlation between drop loss and water capacity in muscle, so drop loss could be used as a quantitative indicator of muscle water capacity [22]. Our results showed lower drop loss in breast and thigh muscle of broilers fed diets supplemented with MO, mainly for the reason that linoleic acid contained in the diets could play an important role in increasing water retention capacity of pectoral muscle [17,23].

Broilers fed MO also showed improved serum parameters. Serum glucose is an important source of energy for animals and can be conducive to growth of body tissues. The increased concentration of serum glucose in our study is associated with increased serum concentrations of insulin and glucagons in broilers fed diets rich in SFA [24], which reveals that MO (containing high content of palmitic acid and SFA) can help to promote glucose metabolism and improve performance of broilers. Serum concentrations of albumin and total protein reflect the functions of synthesis of proteins in liver of broilers, which may associate with animal growth and physiological status [25]. In the present study, we found an increased liver percentage along with an increased concentration of globulin, total protein and albumin, which indicated that MO can improve liver function and performance of broilers [26]. Serum concentration of TC is assessed as a mean of evaluating the lipidaemic status of birds [26]. Our study showed broilers fed MO had a tendency for a decreased concentration of serum TC. A possible mechanism for the lower TC concentration in serum of broilers can be that the rate of triglycerides synthesis in the liver exceeds the capacity to produce very low density lipoproteins, resulting in triglycerides to be deposited in the liver, and hence reduced serum TC [27].

In the present study, we found broilers fed MO showed increased concentration of SFA, which may due to the high concentration of SFA and palmitic acid containing in MO can be deposited in the muscle tissue of broilers [28]. Moreover, oleic acid in MO appears to play a direct role in facilitating the absorption of the SFAs [29]. Studies have demonstrated broilers fed diets rich in SFA showed increased fatty acid deposition in chicken than those fed diets rich in PUFA [2,12]. The MO in our study containing 20% palm oil and 15% linseed oil, palm oil is rich in SFAs and is more stable than linseed oil, while a combination of them would increase stability of the fats and maintenance of an appropriate balance between SFAs and PUFAs [10,30]. Our study also found broilers fed MO1 had decreased concentration of C18:1n-9 and C18:3n-3, as well as increased content of C20:5n-3, C22:6n-3, n-6 PUFA, n-3 PUFA, and total PUFA in breast muscle. Similarly, Wang et al [31] found a combination of linseed and palm oils in chicken diets had enhanced n-3 PUFA content in meat of broilers. These results are likely due to high concentrations of α-linolenic acid (C18:3n-3) in MO can act as a precursor for long chain n-3 PUFA in broilers, such as C20:5n-3 and C22:6n-3, so there are lower content of C18:3n-3 and higher deposition of n-3 PUFA in breast muscle of broilers [7,32]. Broilers fed a diet supplemented with linseed oil can produce chicken meat with higher concentration of n-3 PUFA and lower n-6/n-3 ratio [7], which may benefit human health as a source of n-3 PUFA. A balanced n-6/n-3 PUFA ratios in diets could also help to decrease fat deposition by inhibiting fat synthesis [33]. Our study has demonstrated the beneficial effect of MO on health fatty acid profile of breast muscle in broilers, which may have economic benefits [31].

## CONCLUSION

In conclusion, dietary supplementation with a combination of 15% corn oil, 10% coconut oil, 15% linseed oil, 20% palm oil, 15% peanut oil, and 25% soybean oil had positive effects on ADG, FCR, liver percentage, serum glucose, total serum protein and meat quality, as well as higher SFAs and PUFAs deposition in breast muscle of broilers, which indicates these two mixed plant oils can be used as better dietary energy feedstocks than soybean oil alone.

## Figures and Tables

**Table 1:** Chemical composition of mixed oils (analyzed value, %)

Item	MO11)	MO21)
Dry matter	99.6	95.4
Crude protein	-	4.03
Ether extract	99.0	51.5
Ash	-	5.71
Fatty acids (% of total fatty acid)		
C8:0	0.27	0.12
C10:0	0.22	0.14
C12:0	1.77	0.87
C14:0	1.02	0.53
C16:0	17.4	9.79
C18:0	2.80	1.62
C18:1n-9	17.5	10.2
C18:2n-6	36.3	21.7
C18:3n-3	2.50	1.49
C20:0	0.24	0.14
C22:0	0.23	0.16
C24:0	0.14	0.09

1) MO1, mixed oil 1; MO2, mixed oil 2.

**Table 2:** Composition and nutrient levels of experiment diets (%, as-fed basis)

Item	Phase 1 (d 1 to 21)			Phase 2 (d 22 to 42)		
	
	CTR1)	MO11)	MO21)	CTR1)	MO11)	MO21)
Corn	58.70	58.70	58.70	65.40	65.40	65.40
Soybean meal (44%)	30.11	30.11	30.11	22.69	22.69	22.69
Corn gluten meal (62%)	4.00	4.00	4.00	5.10	5.10	5.10
Soybean oil	3.00	0.00	0.00	3.00	0.00	0.00
Mixed oil 1	0.00	3.00	0.00	0.00	3.00	0.00
Mixed oil 2	0.00	0.00	3.00	0.00	0.00	3.00
Dicalcium phosphate	1.70	1.70	1.70	1.25	1.25	1.25
Limestone	1.39	1.39	1.39	1.44	1.44	1.44
Salt	0.30	0.30	0.30	0.30	0.30	0.30
L-lysine HCl (78%)	0.12	0.12	0.12	0.21	0.21	0.21
DL-methionine (98%)	0.15	0.15	0.15	0.05	0.05	0.05
L-threonine (98%)	0.03	0.03	0.03	0.06	0.06	0.06
Vitamin-mineral premix2)	0.50	0.50	0.50	0.50	0.50	0.50
Nutrient levels						
Gross energy (MJ/kg)3)	16.72	16.47	16.07	16.66	16.45	16.41
Crude protein3)	21.13	21.46	21.51	20.10	20.15	20.16
Calcium4)	0.80	0.80	0.80	0.90	0.90	0.90
Available phosphorus4)	0.45	0.45	0.45	0.35	0.35	0.35
SID lysine	1.10	1.10	1.10	1.00	1.00	1.00
SID methionine	0.50	0.50	0.50	0.38	0.38	0.38
SID threonine	0.80	0.80	0.80	0.74	0.74	0.74
SID tryptophan	0.27	0.27	0.27	0.23	0.23	0.23

SID means standardized ileal digestible.

1) CTR, control, soybean oil; MO1, mixed oil 1; MO2, mixed oil 2.

2) Premix supplied per kg diet: vitamin A, 11,000 IU; vitamin D, 3,025 IU; vitamin E, 22 mg; vitamin K_3_, 2.2 mg; thiamine, 1.65 mg; riboflavin, 6.6 mg; pyridoxine, 3.3 mg; cobalamin, 17.6 μg; nicotinic acid, 22 mg; pantothenic acid, 13.2 mg; folic acid, 0.33 mg; biotin, 88 μg; choline chloride, 500 mg; iron, 48 mg; zinc, 96.6 mg; manganese, 101.76 mg; copper, 10 mg; selenium, 0.05 mg; iodine, 0.96 mg; cobalt, 0.3 mg.

3) Analyzed value.

4) Calculated value.

**Table 3:** Effects of mixed oils on growth performance for broilers

Item	CTR1)	MO11)	MO21)	SEM	p-value
d 0 body weight (g)	44.9	45.1	45.4	0.73	0.87
d 21 body weight (g)	648^b^	722^a^	698^ab^	16.5	0.02
d 42 body weight (g)	2,352^b^	2,543^a^	2,495^a^	32.1	<0.01
d 1 to 21					
Average daily gain (g/d)	28.7^b^	32.2^a^	31.1^ab^	0.79	0.03
Average daily feed intake (g/d)	43.3	45.4	45.4	1.15	0.38
Feed conversion ratio (g/g)	1.51^a^	1.41^b^	1.46^ab^	0.03	0.05
d 22 to 42					
Average daily gain (g/d)	81.1^b^	86.8^a^	85.6^a^	1.42	0.04
Average daily feed intake (g/d)	139	135	136	8.46	0.92
Feed conversion ratio (g/g)	1.72	1.55	1.59	0.09	0.39
d 1 to 42					
Average daily gain (g/d)	54.9^b^	59.5^a^	58.3^a^	0.76	<0.01
Average daily feed intake (g/d)	91.3	90.0	90.5	4.18	0.98
Feed conversion ratio (g/g)	1.66	1.51	1.55	0.06	0.25

SEM means standard error of the mean.

1) CTR, control, soybean oil; MO1, mixed oil 1; MO2, mixed oil 2.

a–b Different superscripts within a row indicate a significant difference (p≤0.05).

**Table 4:** Effects of mixed oils on meat quality and viscera percentages of broilers on d 42

Item	CTR1)	MO11)	MO21)	SEM	p-value
Breast muscle (g)	196	212	214	11.13	0.46
Lightness	38.0	38.1	38.5	0.82	0.92
Redness	3.11	3.17	3.26	0.43	0.94
Yellowness	8.94^c^	7.39^d^	8.53^cd^	0.40	0.06
Drip loss (%)	1.93^a^	0.59^b^	0.49^b^	0.40	0.05
Thigh muscle (g)	233	248	241	6.77	0.34
Lightness	39.1	41.0	40.4	1.18	0.54
Redness	4.40^b^	7.14^a^	6.14^a^	0.48	<0.01
Yellowness	10.0^c^	8.73^d^	10.3^c^	0.47	0.09
Drip loss (%)	0.69^a^	0.30^b^	0.23^b^	0.04	<0.01
Viscera percentages					
Heart	0.63	0.57	0.59	0.03	0.48
Liver	1.96^b^	2.15^b^	2.50^a^	0.09	<0.01
Spleen	0.96	0.98	1.08	0.10	0.69
Pancreas	0.16	0.13	0.15	0.01	0.28
Abdominal fat	0.19	0.19	0.21	0.02	0.57

SEM, means standard error of the mean.

1) CTR, control, soybean oil; MO1, mixed oil 1; MO2, mixed oil 2.

a–b Different superscripts within a row indicate a significant difference (p≤0.05).

c–d Different superscripts within a row indicate a tendency of difference (0.05<p≤0.10).

**Table 5:** Effects of mixed oils on serum parameters of broilers (mmol/L)

Item	CTR1)	MO11)	MO21)	SEM	p-value
d 1 to 21					
Glucose	17.4^d^	18.5^c^	17.4^d^	0.36	0.08
Albumin	33.1	31.0	33.1	1.69	0.60
Globulin	20.5	19.3	20.1	1.22	0.74
Albumin/globulin	0.62	0.69	0.66	0.03	0.21
Total protein	33.1	31.0	33.1	1.69	0.60
Total cholesterol	4.16	3.80	3.69	0.24	0.39
HDL-C	1.78	1.59	1.45	0.10	0.12
d 22 to 42					
Glucose	15.5^b^	18.3^a^	18.8^a^	1.05	0.05
Albumin	12.3^b^	14.3^a^	14.0^a^	0.46	0.04
Globulin	18.4^d^	24.7^c^	22.6^c^	1.66	0.07
Albumin/globulin	0.70	0.59	0.63	0.04	0.20
Total protein	31.1^d^	39.0^c^	36.6^c^	2.03	0.06
Total cholesterol	3.12^c^	2.80^d^	3.12^c^	0.15	0.10
HDL-C	1.28	1.14	1.25	0.08	0.41

SEM, means standard error of the mean; HDL-C, high-density lipoprotein cholesterol.

1) CTR, control, soybean oil; MO1, mixed oil 1; MO2, mixed oil 2.

a–b Different superscripts within a row indicate a significant difference (p≤0.05).

c–d Different superscripts within a row indicate a tendency of difference (0.05<p≤0.10).

**Table 6:** Effects of mixed oils on fatty acid deposition in breast muscle of broilers (g/100 g fresh meat)

Item	CTR1)	MO11)	MO21)	SEM	p-value
C14:0	0.47^c^	0.86^a^	0.68^b^	0.04	<0.01
C16:0	21.8^b^	30.1^a^	27.7^a^	1.19	0.01
C16:1n-7	4.18^d^	2.56^e^	2.85^de^	0.43	0.06
C17:0	0.12^c^	0.25^a^	0.16^b^	0.01	<0.01
C18:0	8.40^c^	17.0^a^	14.5^b^	0.37	<0.01
C18:2n-6	25.6^b^	33.3^a^	24.8^b^	1.02	<0.01
C18:3n-6	0.28	0.37	0.31	0.05	0.34
C18:3n-3	2.02^a^	1.57^b^	1.12^c^	0.09	<0.01
C20:0	0.12^c^	0.22^a^	0.18^b^	0.01	<0.01
C20:1n-9	0.25	0.40	0.36	0.05	0.18
C20:4n-6	2.46^c^	6.15^a^	4.79^b^	0.39	<0.01
C20:5n-3	0.12^b^	0.28^a^	0.17^b^	0.02	<0.01
C22:6n-3	0.30^b^	0.96^a^	0.47^b^	0.13	0.03
SFA^2)^	30.9^c^	48.4^a^	43.2^b^	1.21	<0.01
Total PUFA^2)^	30.8^b^	42.6^a^	31.6^b^	0.82	<0.01
n-6 PUFA	28.4^b^	39.8^a^	29.9^b^	0.80	<0.01
n-3 PUFA	2.44^b^	2.81^a^	1.76^c^	0.09	<0.01
n-6/n-3	11.7^c^	14.2^b^	17.2^a^	0.64	<0.01
P/S^2)^	1.00^a^	0.88^b^	0.73^c^	0.01	<0.01

SEM means standard error of the mean; SFA, saturated fatty acids; PUFA, polyunsaturated fatty acids; P/S: polyunsaturated fatty acids/saturated fatty acids ratio.

1) CTR, control, soybean oil; MO1, mixed oil 1; MO2, mixed oil 2.

a–c Different superscripts within a row indicate a significant difference (p≤0.05).

d–e Different superscripts within a row indicate a tendency of difference (0.05<p≤0.10).
